# Non-invasive methods for embryo selection

**Published:** 2016-06-27

**Authors:** HN Sallam, NH Sallam, SH Sallam

**Affiliations:** Department of Obstetrics and Gynaecology, the University of Alexandria in Egypt.; Alexandria Fertility Center, Alexandria, Egypt.

**Keywords:** Embryo selection, non-invasive, metabolomics, sHLA-G, oxidative stress, morpho-kinetics, time lapse monitoring

## Abstract

With the widespread use of assisted reproduction, a simple and practical method for embryo selection is needed to optimize the chances of pregnancy while diminishing the incidence of multiple pregnancy and its accompanying problems. Many non-invasive methods for embryo selection have been proposed and some are more promising than others. This review summarizes these methods and attempts to evaluate them in the light of the best currently available evidence and to find out whether any of them is ripe for replacing or supplementing the time-honored method of morphological assessment.

Despite continuous developments in the field of assisted reproduction, the live birth rate following IVF and ICS remains low. The most recent statistics of the Society for Assisted Reproductive Technologies (SART) of the American Society for Reproductive Medicine (ASRM) show that the live birth rate in women undergoing these procedures in 2011 was 29.2% per retrieval (SART, 2014). In order to maximize the success rates, infertility specialists are always faced with the dilemma of transferring too many embryos and risking the occurrence of multiple pregnancies with the resulting complications, or transferring too few embryos and risking failure of the procedure (El-Toukhy et al., 2006). There is, therefore, an urgent need for a simple and practical method for embryo selection in order to maximize the chances of success and minimize the problems of multiple pregnancies in couples treated with assisted reproduction.

Various methods for embryo selection have been suggested and practiced. These include invasive and non-invasive methods. Invasive methods are used to screen the embryos for genetic abnormalities (PGS). They require the performance of an embryo biopsy: a blastomere biopsy at the 8-cell stage or a trophectoderm biopsy at the blastocyst stage. The procedures are expensive and require advanced skills. More importantly, they are not full proof due to the possibility of embryo injury, undetected mosaicism and segmental aneuploidy (Juneau et al., 2016). This means that some normal embryos may be discarded and some abnormal embryos may be falsely diagnosed as normal. In addition, a meta-analysis of randomized studies conducted in 2011 showed that PGS did not improve the live birth rate compared to morphological selection (Mastenbroek et al., 2011), although a more recent meta-analysis showed that, in good prognosis patients, comprehensive chromosome screening after blastocyst biopsy was associated with higher clinical implantation and ongoing pregnancy rates (but not higher live birth rates) when the same number of embryos is transferred (Dahdouh et al., 2015). Consequently, more infertility specialists are now considering and using non-invasive methods for embryo selection. The aim of this paper is to evaluate these methods in the light of evidence.

## Non-invasive methods for embryo selection

Non-invasive methods for embryo selection include (1) selection on the basis of the morphology of individual embryos at the time of transfer, (2) selection on the basis of the morpho-kinetic changes of individual embryos during early developmental stages observed by time-lapse photography, (3) selection on the basis of the oxygen consumption by individual embryos, (4) selection on the basis of various biochemical markers measured in the culture medium of individual embryos, (5) selection on the basis of oxidative stress to which individual embryos are subjected, or (6) a combination of some of the above methods.

## Evaluation of methods for embryo selection

Evaluation of methods for embryo selection is a three-step exercise. In an ideal world, the first step should involve the retrospective comparison of the method in pregnant versus non-pregnant patients and finding a statistically significant difference between both groups. The second step requires the construction of receiver-operator characteristic (ROC) curves in order to determine the cut-off point at the best sensitivity and specificity. The third step requires conducting prospective randomized trials using this cut-off point to determine the accuracy and applicability of the method with the live birth rate as the primary end point. However, the occurrence of pregnancy does not only depend on choosing the embryo(s) with the best potential for implantation, as the process is confounded by other steps involved in assisted reproduction, namely the difficulties encountered during embryo transfer, the receptivity of the endometrium, the presence of intra-uterine abnormalities as well as any systemic disease affecting implantation. Consequently, the development of healthy-looking blastocysts is sometimes taken as the end-point of the study instead of the clinical outcome.

## Embryo selection on the basis of morphology

Classically, embryos were and still are selected for transfer on the basis of their morphology. Various scoring systems were developed to evaluate the embryos at various stages of development: in the pro-nuclear stage, in the cleavage stage or in the blastocyst stage.

### Scoring at the 2-PN stage

**Fig. 1:**
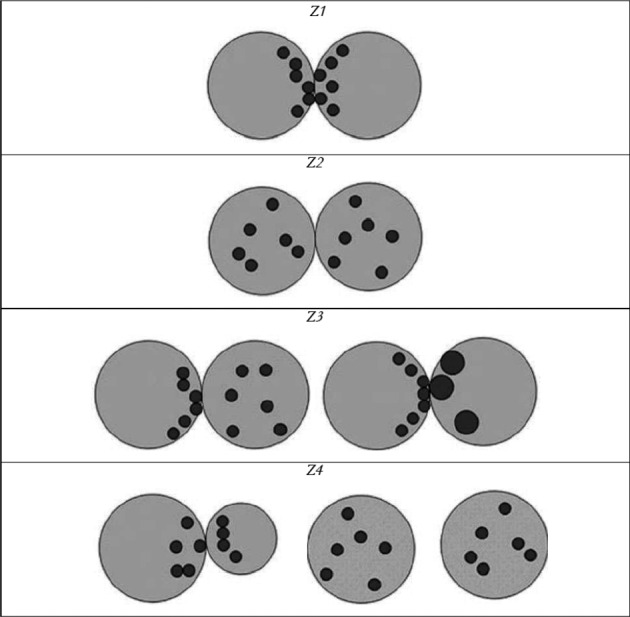
— Embryo scoring at the pronuclear stage (Z1 = equal pronuclei, equal number and size of nucleoli, aligned in both pronuclei at the pronuclear junction, number of nucleoli 3 to 7; Z2 = equal pronuclei, equal number and size of nucleoli, scattered in both pronuclei, number of nucleoli 3 to 7; Z3 = equal pronuclei, equal number and even or (and) uneven size of nucleoli, aligned in one pronucleus at the pronuclear junction, number of nucleoli 3 to 7; Z4 = unequal or separated pronuclei) (from Scott et al., 2000).

In 2000, Scott and co-workers developed a scoring system for embryos in the pronuclear stage (Scott et al., 2000). Scoring is performed at 16-18 hours after fertilization and is based on the following criteria: (1) size of the pronuclei and their symmetry, (2) size, number, equality and distribution of the nucleoli and (3) the appearance of the cytoplasm (Fig. 1). In a retrospective study, Balaban et al. found that embryos that showed an ideal pronuclear pattern (0 PN pattern) cleaved earlier and faster and resulted in better quality cleavage stage embryos and blastocysts. They also found that blastocysts derived from zygotes with a high 2PN stage score have a higher potential for implantation (Balaban et al., 2001). Similar results were reported by Ludwig et al. (2000) and Zollner et al. (2002) who constructed ROC curves and used a score of 15 as their cut-off value. In a different study, Kahraman et al. (2002) found that pre-embryos with a low 2PN score had a low rate of blastocyst formation and, more importantly, a high risk of chromosomally abnormal embryos. However, in 2014, Berger et al. challenged these findings. They compared the clinical pregnancy rate (CPR) resulting from the transfer of 835 non-PN scored embryos with 1064 PN scored embryos and found that PN scoring was not associated with improved CPR in day 3 embryo transfers (Berger et al., 2014).

### Scoring at the cleavage stage

Scoring embryo morphology at the cleavage stage is more commonly used. The scoring systems are largely based on two studies: Giorgetti et al. (1995) and Van Royen et al. (1999).

In the Giorgetti study, a total of 957 single embryo transfers were carried out 48 hours after insemination. The authors found that the embryos with the best potential for implantation were those who (1) had cleaved, (2) had no fragmentation, (3) displayed no irregularities, and (4) had reached the 4 cell stage by 48 hours after insemination (Giorgetti et al., 1995). Similarly, Van Royen et al. (1999) performed 23 double transfers resulting in ongoing twins. They found that the criteria associated with embryo implantation were (1) the absence of multi- nucleated blastomeres, (2) the presence of 4 or 5 blastomeres on day 2, (3) the presence of 7 or more cells on day 3 and (4) </= 20% anucleated fragments.

**Fig. 2:**
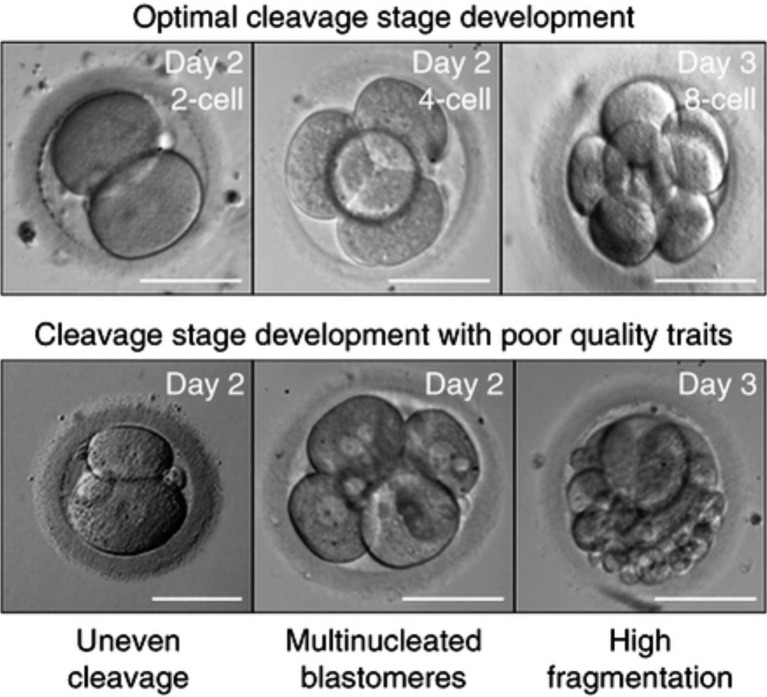
— Grading the embryo at the cleavage stage (from O’Leary et al., 2013)

These studies were in line with the grading system first suggested by Lucinda Veeck in 1991 (Veeck, 1991) (Table I). Hsu et al. (1999) used this scoring system to grade embryos resulting from IVF and ICSI and found that it was a valuable, although limited, indicator of implantation outcome. A different scoring system was also devised by Houghton et al. (2002) using similar criteria (Table II). Examples of cleavage embryos by Oleary et al. (2013) are shown in Figure 2. We have recently conducted a study comparing the predictive value of soluble human leuckocytic antigen-G (sHLA-G) concentration in culture medium to the Veeck’s score and found that the Veeck’s score was a better predictor of implantation. The ROC curve revealed an area under the curve of 0.925 for the Veeck’s score compared to 0.794 for the s-HLA-G (Sallam et al., 2015).

**Table I:** — Cleavage stage embryo scoring (day 3) (from Veeck, 1991).

**Grade**	**Description**
Grade 1	Embryo with blastomeres of equal size, no cytoplasmic fragments
Grade 2	Embryo with blastomeres of equal size, minor cytoplasmic fragments or blebs
Grade 3	Embryo with blastomeres of distinctly unequal size, none or few cytoplasmic fragments
Grade 4	Embryo with blastomeres of equal or unequal size, signi cant cytoplasmic fragmentation
Grade 5	Embryo with few blastomeres of any size, severe or complete fragmentation

**Table II:** — Cleavage stage embryo scoring (day 3) (from Houghton et al., 2002).

**Embryo grade**	**Classification**
Grade 1	Even blastomeres, no fragmentation
Grade 2	Even blastomeres, < 10% fragmentation
Grade 2.5	10-30% fragmentation
Grade 3	> 30% fragmentation
Grade 4	Only one intact blastomere
Grade 5	No viable blastomeres

### The graduated embryo score (GES)

Fish et al. (2001) took the matter further and introduced the graduated embryo score (GES) where the embryos are scored on 3 successive occasions: at 16-18 hours, then at 25-27 hours and again at 64-67 hours. The total score out of a maximum of 100 is then calculated as shown in table III. In their study, they found that embryos which scored > 70 had an implantation rate of 39% compared to 24% for embryos that scored < 65 (Fish et al., 2001).

**Table III:** — Graduated embryo score (from Fisch et al., 2001).

**Evaluation**	**Hours after insemination**	**Developmental Mileston**	**Score**
1	16-18	Nucleoli aligned	20
2	25-27	Cleavage regular and symmetrical	30
		Fragmentation absent	30
		Fragmentation < 20%	25
		Fragmentation > 20%	0
3	64-67	Cell number and grade 7, I; 8, I; 8, II; 9, I	20
		7, II; 9, II; 10, I; 11, I; compacting I	10
		Total score			100

### Staging at the blastocyst stage

**Fig. 3:**
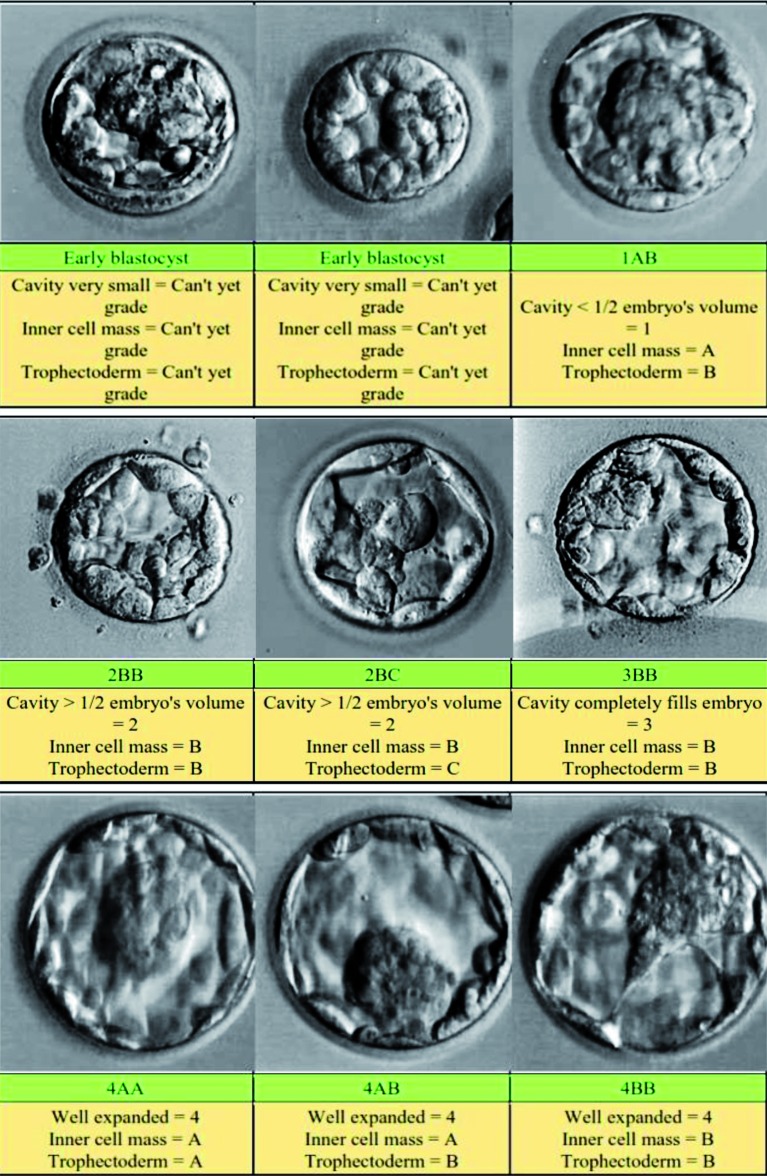
— Scoring the embryo at the blastocyst stage (from Gardner et al., 2000)

A method for grading embryos at the blastocyst stage was devised by Gardner et al. (2000). The score is based on 3 criteria (1) the blastocyst development and stage status, (2) the inner-cell mass quality and (3) the trophectoderm quality (Fig. 3 and Table IV). In their study they transferred 2 embryos at the blastocyst stage in each patient. They found that when they replaced 2 top grade blastocysts (> 3AA), the implantation and clinical pregnancy rates were 72.8% and 86.8%, respectively, compared to 54.3% and 69.6% when one blastocyst only was top grade and to 28.1% and 43.8% when both blastocysts were less than top grade quality (< 3AA) (Gardner et al., 2000). Blastocyst transfer is now being increasingly used in assisted reproduction as it provides a method of natural selection of embryos when a large number of these are available. The most recent Cochrane review has shown that there is a small significant difference in live birth rates in favour of blastocyst transfer (Day 5 to 6) compared to cleavage stage transfer (Day 2 to 3) (OR = 1.40; 95% CI = 1.13 to 1.74). However, cumulative clinical pregnancy rates from cleavage stage (derived from fresh and thaw cycles) resulted in higher clinical pregnancy rates than from a single blastocyst cycle (OR = 1.58; 95% CI = 1.11 to 2.25) (Glujovsky et al., 2012).

**Table IV:** — Scoring the embryo at the blastocyst stage (from Gardner et al., 2000).

**Expansion grade**	**Blastocyst development and stage status**
1	Blastocoel cavity less than half the volume of the embryo
2	Blastocoel cavity more than half the volume of the embryo
3	Full blastocyst, cavity completely filling the embryo
4	Expanded blastocyst, cavity larger than the embryo, with thinning of the shell
5	Hatching out of the shell
6	Hatched out of the shell
ICM grade	Inner cell mass quality
A	Many cells, tightly packed
B	Several cells, loosely grouped
C	Very few cells
TE grade	Trophectoderm quality
A	Many cells, forming a cohesive layer
B	Few cells, forming a loose epithelium
C	Very few large cells

### Computerized embryo selection

In an attempt to automate embryo selection, our group collaborated with Dr. Claudio Manna’s group in devising a computerized method for embryo selection based on character recognition. Each embryo picture is transformed into a vector and the computer trained to recognize embryos with the best potential for implantation. The results showed that the ability of the computer to recognize the best embryos was comparable to that of a group of experts (Manna et al., 2004). However, the method has not yet been taken to the prospective randomized trial phase.

## Embryo selection on the basis of morpho-kinetics

Embryos can also be selected on the basis of their morpho-kinetic changes. It has been shown from the outset of IVF work that earlier cleaving embryos have a better chance of developing into blastocysts and implanting (Edwards et al., 1984; Shoukir et al., 1997; Salumets et al., 2003; van Montfoort et al., 2004). Payne et al. (1997) and Mio and Maeda (2008) were also among the first groups to describe the dynamic changes occurring in the embryo from the time of fertilization until blastocyst formation using time-lapse photography.

Based on these and other observations, automatic systems were developed to record the dynamic changes of cultured embryos using a time-lapse camera placed inside the incubator. Some of the currently available systems consist of an incubator with a built-in camera, while others consist of cameras that can be placed inside traditional CO2 incubators. Some systems use bright field technology while the others use dark field technology (which is associated with a better view of the membranes but a lower overall resolution). The accompanying software is meant to help the embryologist to choose the embryo(s) with the best potential for implantation. This software is based upon retrospective studies that identified the morpho-kinetic changes associated with the best blastocyst formation, the highest implantation, pregnancy or take home baby outcome. Table V summarizes the main characteristics of 3 commercially available systems (Kovacs, 2014).

**Table V:** — Technical parameters of three commercially available time-lapse systems (from Kovacs, 2014).

	**Embryoscope**	**Primo vision**	**EEVA**
Illumination	Bright field, low intensity red LED	Bright field, low intensity green LED	Dark field
Microscope incubator	Incubator with integrated time- lapse system	Microscope that can be placed in standard incubators	Microscope that can be placed in standard incubators
Culture dish	Embryoslide	9-16 well Primo vision embryo culture dish	EEVA dish
Embryo culture	Single culture	Group culture	Group culture
Planes of view	7 focal planes	11 focal planes	Single plane
Max no. of embryos	72	96	Depends on the dish
Other	Comes with software	Comes with software	Automated, software scores blastocyst formation potential

Using one of these systems, Meseguer et al. (2011) conducted a retrospective time-lapse video observational study of 247 embryos with implantation as their end point. They found that (1) direct cleavage from 1 to 3 cells (very short time as a 2-cell embryo), (2) uneven blastomeres at the 2-cell stage and (3) multinucleation at the 4-cell stage were associated with a minimal chance of implantation and considered them exclusion criteria. On the other hand, they found that the best three parameters predictive of implantation were (1) cleavage to the 5-cell stage called t5, (2) time from 3 to 4 cell division called S2 and (3) time from 2 to 3 cell division called CC2 (Meseguer et al., 2011). The optimal ranges for t5, S2 and CC2 were 48.8-56.6 hrs, ≤ 0.76 hrs and ≤ 11.8 hrs, respectively.

Other groups, using the same or different systems, found slightly different predicting parameters with different ranges (Table VI) (Wong et al., 2010; Cruz et al., 2012; Conaghan et al., 2013). The association between time-lapse markers of embryo development and embryo aneuploidy was also studied by time- lapse systems with contradictory results (Table VII) (Chavez et al., 2012; Campbell et al., 2013; Basile et al., 2014).

**Table VI:** — Optimal time interval of kinetic markers predictive of implantation or blastocyst formation by various groups.

	**Wong 2010**	**Meseguer 2011**	**Cruz 2012**	**Conaghan 2013**
End-point	Blastocyst	Implantation	Blastocyst	Blastocyst
S _1_ (min)	14.3 ±			
CC _2_ (hour)	11.1 ± 2.2	≤ 11.9		9.3-11.5
S _2_ (hour)	1 ± 1.6	≤ 0.76	≤ 0.76	≤ 1.73
t _2_ (hour)		48.8-56.6	48.8-56.6	

**Table VII:** — Optimal time interval of kinetic markers predictive of embryo euploidy by various groups.

	**Basile et 2014**	**Chavez et 2012**	**Campbell 2013**
S _1_ (min)		14.4 ± 4.2	
CC _2_ (hour)		11.8 ± 0.7	
S _2_ (hour)		0.96 ± 0.84	
t _5_ (hour)	47.2-58.2		
t_5-2_ (hour)	< 20.5		
CC_3_ (hour)	11.7-18.2		
t_BC_ (hour)			< 122.9

In 2014, Rubio et al. conducted a randomized controlled trial (RCT) to evaluate the time-lapse monitoring system (TMS). They found that the system improved the implantation and on-going pregnancy rates and decreased the miscarriage rate significantly (Rubio et al., 2014).

However, these results could not be confirmed by a more recent RCT of 235 patients conducted by Goodman et al. who found that the addition of time-lapse morpho-kinetic data did not significantly improve clinical reproductive outcomes in all their patients including those that reached the blastocyst stage (Goodman et al., 2016). In addition, the latest Cochrane review shows that the time-lapse monitoring systems did not improve the live birth rate or the clinical pregnancy rate (OR = 1.11, 95% CI = 0.45-2.73 and OR = 1.23, 93% CI = 0.96-1.59, respectively) (Armstrong et al., 2015).

## Embryo selection on the basis of the oxygen consumption

Oxygen consumption by individual embryos was studied as a potential method for selecting the embryos with best developmental capability (and hence implantation) and various methods for its measurement were described. In 1996, Houghton et al. used a non-invasive ultra- microfluorescence technique to measure oxygen consumption by pre-implantation and early post- implantation mouse embryos (Houghton et al., 1996). Oxygen consumption was detected at all stages of development, including in the early post-implantation embryo. Consumption remained relatively constant from zygote to morula stages before increasing in the blastocyst and day 6.5-7.5 stages (Houghton et al., 1996). Similar results were also reported by the same group for bovine embryos grown in culture (Thompson et al., 1996). In 2000, Trimarchi et al. used the self-referencing electrode technique to measure gradients of dissolved oxygen in the culture medium immediately surrounding developing mouse embryos. They found a two-fold increase in oxygen consumption at the blastocyst stage. They also found that mitochondrial oxidative phosphorylation accounted for 60-70% of the oxygen consumed by blastocysts, while it accounted for only 30% of the total oxygen consumed by cleavage-stage embryos (Trimarchi et al., 2000). More recently, a respirometric biochip was developed for measuring oxygen consumption and used for embryo assessment (O’Donovan et al., 2006).

In 2012, Tejera et al. used the Clark O_2_ sensor embedded in a time-lapse morphology system to study oxygen consumption by individual human embryos (Lopes et al., 2007). The system allows the continuous measurement of individual respiration rates with simultaneous acquisition of digital images of each embryo, during the entire culture period (6-7 days).

Tejera et al. found that embryo oxygen consumption in women who became pregnant was significantly higher compared to those who did not. The authors constructed ROC curves for oxygen consumption and reported a cut-off value of 5.89 fmol per second at the optimum sensitivity and specificity of 55.6% and 87.5%, respectively (Tejera et al., 2012). These preliminary data require further evaluation by prospective randomized trials in single embryo transfer cycles with the live birth rate at their primary end point before this expensive method can be suggested for use in routine clinical practice.

## Embryo selection on the basis of biochemical markers in the culture medium

The metabolism of the pronucleate oocyte, the cleavage stage embryo and the blastocyst has been thoroughly studied in vitro as elegantly reviewed by Gardner and Wale (2013). Some substrates present in the culture medium are consumed by the embryo while others metabolites are secreted. The measurement of some of these products in the culture medium has therefore been suggested as a method for embryo selection. These include (1) substrates and products of carbohydrate metabolism (2) amino acid turnover (3) assessment of the embryo metabolome (4) measurement of the s-HLA-G fragment (5) ßßHCG (6) haptoglobin-α-1 fragment (7) leptin (8) ubiquitin and (9) platelet activating factor (PAF) concentrations in the culture medium.

### Substrates and products of carbohydrate metabolism

During the first 2 days following fertilization, embryos use pyruvate preferentially as their main source of energy, while after compaction they start to consume glucose as their main source (Leese et al., 1986; Gardner and Leese, 1987; Gott et al., 1990; Jones et al., 2001).

In 1980, Renard et al. were the first to show that cattle embryos with a glucose uptake of >5 mg/h developed better both in vitro and in utero than those embryos with a lower uptake (Renard et al., 1980). Subsequently, Gardner and Leese reported similar results in mouse blastocysts and in 2001, Gardner et al. showed that the utilization of pyruvate and glucose on day 4 was predictive of blastocyst development in human embryos (Gardner and Leese, 1987; Gardner et al., 2001). Gardner et al. took the matter further and showed, in a study using single embryo transfers in humans, that glucose consumption was also predictive of pregnancy (Gardner et al., 2011). It was also shown that embryos that gave rise to pregnancy were not necessarily those who had the highest morphology scores and that human female embryos consumed more glucose compared to male embryos (Gardner et al., 2011). Time has come to conduct a prospective randomized study to evaluate the applicability and reliability of this method of embryo selection.

### Amino acid turnover

Embryos grown in vitro consume some amino acids present in the culture medium and produce (secrete) others. This amino acids turn-over was evaluated as a method for embryo selection. In 2002, Houghton et al. used high performance liquid chromatography (HPLC) to study the amino acid turn-over in the culture medium of individually-grown human embryos. They found that embryos that went on to develop top quality blastocysts consumed more leucine and produced more alanine compared to those that did not. The profiles of alanine, arginine, glycine, methionine and asparagine flux predicted blastocyst formation in >95% of instances (Hougthon et al., 2002) (4). In a subsequent study, Brison et al. showed that the turnover of three amino acids, asparagine, glycine and leucine, was significantly correlated with clinical pregnancy and live birth (Brison et al., 2004). More recently, Picton et al. investigated the relationship between amino acid turn-over in the human embryo culture medium and chromosomal anomalies of the embryo (13, 18, 21 X and Y). They found that asparagine, glycine and valine turnover was significantly different between genetically normal and abnormal embryos on days 2 to 3 of culture. By days 3 to 4, the profiles of serine, leucine and lysine differed between euploid versus aneuploid embryos. They also found that cleavage stage male embryos consumed significantly more tryptophan, leucine and asparagine compared to female embryos (P < 0.05) (Picton et al., 2010). To the best of our knowledge, no prospective randomized study has so far been conducted to evaluate the application of data from these studies for embryo selection.

**Fig. 4:**
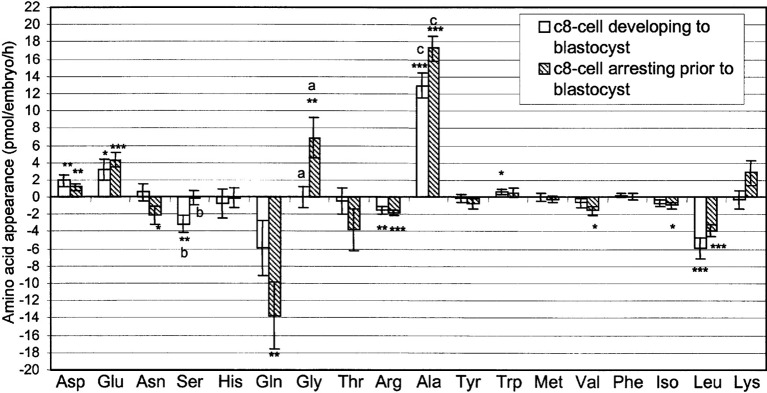
— Amino acid depletion/appearance ± SEM by individual human embryos over the compacting 8-cell to morula transition which subsequently formed blastocysts (n = 23), or which arrested in culture prior to cavitation (n = 32). *P < 0.05; **P < 0.01; ***P < 0.001 significance from zero (from Houghton et al., 2002).

### Assessment of the embryo metabolome

Metabolomic profiling of spent embryo culture media has also been proposed as a method for embryo selection. In this method, an overall metabolic footprint of the surrounding medium is determined, rather than measuring specific nutrients and metabolites (Hollywood et al., 2006). Metabolomic profiling of culture medium can be determined by non-optical spectroscopy such as nuclear magnetic resonance (NMR), mass spectroscopy (MS) with or without high performance liquid chromatography (HPLC) which are costly techniques that require trained personnel and expensive equipment. More commonly, they have been determined using optical spectroscopy such as near infrared (NIR) spectroscopy or Raman spectroscopy, which are less expensive, less complex and do not need sample preparation or separation.

**Fig. 5:**
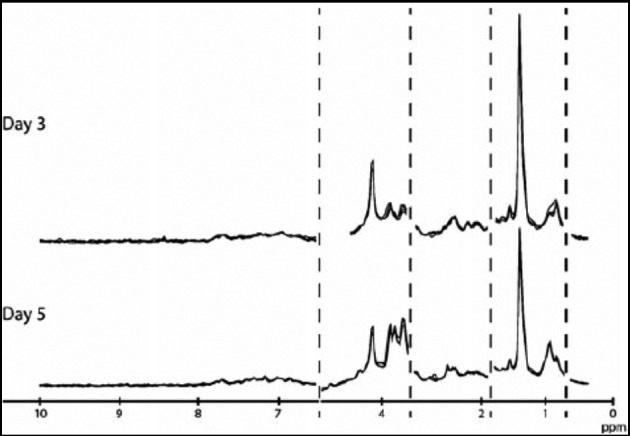
— Spectra of embryo culture media after day 3 and day 5 transfers (from Kirkegaard et al., 2014).

Several studies have been conducted using NIR spectroscopy with the derivation of a “viability score”. The viability score is obtained by comparing regions within the NIR spectrum that discriminate between embryos, which have successfully implanted and those that did not (Fig. 5) (Kirkegaard et al., 2014). These studies reported a good correlation between a high viability score and the ability of the embryo to implant but not with embryo morphology (Seli et al., 2007; Nagy et al., 2008; Scott et al., 2008; Vergouw et al., 2008; Nagy et al., 2009; Seli et al., 2010; Ahlstrom et al., 2011; Seli et al., 2011; Vergouw et al., 2011). However, randomized studies failed to confirm that this method can be used to prospectively select embryos with the best implantation or pregnancy potential as shown in a recent meta-analysis (OR = 0.98; 95% CI = 0.74-1.29) (Vergouw et al., 2014).

### s-HLA-G fragment

The human leukocytic antigen (HLA) system consists of a group of genes that encode for proteins on the surface of cells responsible for regulation of the immune system in humans. This group of genes resides on chromosome 6 and can be considered the human versions of the major histocompatibility complex (MHC) genes found in most vertebrates.

The proteins encoded by these genes are also known as “antigens”, because they were originally found to play an important role in organ transplantation. Different HLA antigens play different roles in the human body but HLA-G is secreted mainly by the extravillous trophoblast at the foetal-maternal interface and plays an important role in the process of implantation by opposing the action of natural killer (NK) cells (Goldman-Wohl et al., 2000; Roussev and Coulam, 2007). Consequently, the concentration of the soluble moiety of the HLA-G fragment (s-HLA-G fragment) was also measured in the spent culture medium of human embryos as a possible method of embryo selection.

Various studies have shown that the concentration of sHLA-G fragment was significantly higher in the spent culture medium of embryos that implanted versus those that did not (Kotze et al., 2013; Guo et al., 2013). This was confirmed in a meta-analysis conducted by Vercammen et al. (2008) who found that the diagnostic odds ratio of predicting a clinical pregnancy was significantly higher when this method was used (DOR = 4.38; 95% CI = 2.93-6.55). Other studies confirmed these results in subsequent studies (Guo et al., 2013; Kotze et al., 2013; Sallam et al., 2016). Despite these encouraging findings, prospective randomized trials in single embryo transfer cycles need to be conducted before the method can be reliably used in a wider clinical context.

### ßßHCG

In 2011, Butler et al. reported that ßßHCG was detectable in the spent culture medium of human embryos from the 2 pronuclear (2PN) stage through to the blastocyst stage (Butler et al., 2011). Similar results were reported by Ramu et al. (2011) on day 2 spent embryo culture media. More recently, Xiao-Yan et al. measured ßßHCG in the spent culture media of human embryos using a highly sensitive electro-chemiluminescence immunoassay. The authors found a positive correlation between ßßHCG concentration and the blastocyst morphological grading (r = 0.411), as well as with the implantation rate (r = 0.559 at day3 and 0.535 at day5) (Xiao-Yan et al., 2013). These interesting observations need to be confirmed by more studies before being evaluated in prospective randomized trials.

### Other bio-markers

Other bio-markers have been studied in the spent culture medium of human embryos as possible markers for embryo selection. These include platelet activating factor (PAF), leptin, ubiquitin and haptoglobin-α-1 fragment.

The platelet activating factor (PAF) also known as the phospholipid 1-o-alkyl-2-acetyl-sn-gylcero-3-phosphocholine is a soluble factor produced by mammalian pre-implantation embryos. Besides causing platelet activation, it influences changes in the oviductal, endometrial and maternal immune functions. It also acts as a trophic survival factor for the early embryo and this has been shown by the in vitro supplementation of culture media with PAF (O’Neill and Saunders, 1984; O’Neill, 2005). In 2002, Roudebush et al. analysed PAF in the spent culture media of human embryos and found a good correlation between PAF levels and pregnancy outcome (Roudebush et al., 2002). Leptin was also measured in the spent culture medium of human embryos by Gonzàlez et al. (2000) who found that human blastocysts secrete it in significantly higher levels compared to arrested embryos.

With more sophisticated equipment available, Katz-Jaffe et al. used time-of-flight mass spectroscopy to analyse the spent culture medium of human and mouse embryos. They found that the protein biomarker ubiquitin was significantly up-regulated (P < 0.05) in embryos that went on to develop blastocysts (Katz-Jaffe et al., 2006). In an equally interesting study, Montskó et al. analysed the spent culture medium of human embryos by liquid chromatography coupled with mass spectroscopy. They identified a marker known as the haptoglobin-α-1 fragment, a polypeptide which was present in higher concentrations in the culture medium of non-viable embryos compared to viable embryos and those who went on to achieve pregnancy (Montskó et al., 2015). They suggested the use of this marker for the exclusion of embryos with low implantation potential. However, the applicability and accuracy of these four methods to select the embryo with the best implantation potential need to be evaluated by prospective randomized trials.

## Embryo selection on the basis of oxidative stress

A relationship between oxidative stress in the culture medium and the growing embryos has been demonstrated. In 1996, Paszkowski and Clarke found that impaired embryo development was associated with an increased generation of reactive oxygen species by the embryo (Paszkowski and Clarke, 1996) and Lipari et al. showed that the mean nitric oxide (a pro-oxidant) metabolite levels in the day 5 culture medium were 2.6 times higher in embryos that progressed to the blastocyst stage than in those that did not (Lipari et al., 2009). The addition of certain nutrients known to be antioxidants to the culture media has also been shown to improve the oxidative state of the early embryo. For example, Ozawa et al. showed that the addition of glutathione or thioredoxin (antioxidant pathways) to the culture medium of porcine embryos reduces the intracellular redox status of these embryos resulting in improved development to the blastocyst stage (Ozawa et al., 2006). Similarly, Suzuki et al. found that the addition of glutamine and hypotaurine to the culture medium of porcine pre-implantation embryos improves their intracellular oxidative status and in- vitro development (Suzuki et al., 2007).

Various systems for the measurement of oxidative stress exist. They include thermo- chemiluminescence (TCL) analyser systems which measure the TCL amplitude in counts per second (CPS) as well as systems based on electrochemical technology that measure the static oxidation reduction potential (sORP) in millivolts (mV) (Shnizer et al., 2003). To test the method for embryo selection, Wiener-Megnazi et al. performed 77 transfers of embryos cultured individually for 72 hours and measured the oxidative status using the thermo-chemiluminescence (TCL) analyser. The mean number of transferred embryos was 2.09 ± 0.65 per transfer. Two parameters were recorded: the TCL amplitude after 50 seconds (H1) and the TCL ratio. They found that the maximal intra-cohort TCL H1 amplitude was significantly lower in women who became pregnant compared to those who did not (P < 0.05). They constructed ROC curves and found that the maximal intracohort TCL H1 cut- off level for the TCL amplitude was 210 counts per second. The combination of maximal intracohort H1 level < 210 counts per second with a TCL ratio of =< 80% had a positive predictive value of 70.6% for the occurrence of pregnancy (Wiener-Megnazi et al., 2011). These promising data need to be confirmed by prospective randomized studies conducted in single embryo transfer cycles using various systems for measuring oxidative stress before the method can be put into wider clinical practice.

## Embryo selection using a combination of markers

Combining two markers has been tried to increase the predictive value of each marker used alone. Kotze et al. (2013) found that combining sHLA-G prediction with the GES, increased the odds ratios for predicting biochemical, clinical and ongoing pregnancies by 2.62 (95% CI = 2.14-3.22), 2.72 (95% CI = 2.22-3.33) and 3.56 (95% CI = 2.88-4.40), respectively. We have also combined the predictive value of sHLA-G fragment and the GES and found that this combination had a predictive value for clinical pregnancy superior to each marker alone. ROC curves showed an area under the curve (AUC) of 0.968 when both markers were combined compared to 0.794 and 0.925 when sHLA-G or GES were used alone, respectively (Sallam et al., 2016).

## Conclusions

Various biophysical, biochemical and physico- chemical markers of embryo development have been identified showing a significant difference between embryos resulting in implantation and pregnancy and those that did not. Some of them are more promising than others as methods of embryo selection in assisted reproduction e.g. glucose consumption by the growing embryos, sHLA-G concentration in the spent medium, measurement of oxidative stress to which individual embryos are subjected and time lapse study of embryo morpho- kinetics (Table VIII). However none of these markers has so far proven its superiority over the others (Table IX). Prospective randomized studies are still needed using individually cultured embryos in cycles with single embryo transfers to identify the marker (or combination of markers) that can be used reliably for this purpose. Until this is achieved, the time honoured morphological scoring remains, so far, the best available method for embryo selection.

**Table VIII:** — Summary of selected studies evaluating various methods of embryo selection.

**Method of embryo selection**	**Outcome studied**	**Statistical difference found**	**Reference**
Scoring at 2-PN stage	Blastocyst formation	Yes	Balaban et al., 2001; Ludwig et al., 2000; Zollner et al., 2002
	Clinical pregnancy	No	Berger et al., 2014
	Euploidy	Yes	Kahraman et al., 2002
Scoring at the cleavage stage	Implantation	Yes	Hsu et al., 1999; Sallam et al., 2015
Graduated embryo score (GES)	Implantation	Yes	Fish et al., 2001
Staging at the blastocyst stage	Implantation	Yes	Gardner et al., 2000
	Clinical pregnancy	Yes	Gardner et al., 2000
Morpho-kinetics by time-lapse systems	Implantation	Yes	Meseguer et al., 2011
	On-going pregnancy	Yes	Rubio et al., 2014
Oxygen consumption	Clinical pregnancy	Yes	Tejera et al., 2012
Pyruvate in culture medium	Blastocyst formation	Yes	Gardner and Leese, 1987; Gardner et al., 2001
Glucose in the culture medium in culture medium	Blastocyst formation	Yes	Gardner and Leese, 1987; Gardner et al., 2001
	Clinical pregnancy	Yes	Gardner et al., 2011
Amino acid turnover in culture medium	Blastocyst formation	Yes	Houghton et al., 2002
	Clinical pregnancy and live birth	Yes	Brison et al., 2004
	Euploidy	Yes	Picton et al., 2010
Embryo metabolome in culture medium	Implantation	Yes	Seli et al., 2007; Nagy et al., 2008; Scott et al., 2008; Vergouw et al., 2008; Nagy et al., 2009; Seli et al., 2010; Ahlström et al., 2011; Seli et al., 2011; Vergouw et al., 2011
s-HLA-G in culture medium	Implantation	Yes	Vercammen et al., 2008; Guo et al., 2013; Kotze et al., 2013; Sallam et al., 2016
HCG in culture medium	Blastocyst formation	Yes	Xiao-Yan et al., 2013
	Implantation	Yes	Xiao-Yan et al., 2013
Platelet activating factor (PAF) in culture medium	Clinical pregnancy	Yes	Roudebush et al., 2002
Leptin in culture medium	Blastocyst formation	Yes	Gonzàlez et al., 2000
Ubiquitin in culture medium	Blastocyst formation	Yes	Katz-Jaffe et al., 2006
Haptoglobin-α-1 fragment in culture medium	Clinical pregnancy	Yes	Montskó et al., 2015
Oxidative stress	Clinical pregnancy	Yes	Wiener-Megnazi et al., 2011

**Table IX:** — Summary of meta-analyses of prospective randomized studies to evaluate the proposed method of embryo selection.

**Method of embryo selection**	**Outcome studied**	**Statistical difference found**	**Odds ratio (95% confidence intervals)**	**Reference**
Scoring at the blastocyst stage	Live birth	Yes, if equal number of embryos are transferred	1.40 (95% CI = 1.13-1.74)	Glujovsky et al., 2012 (Cochrane review)
	Cumulative pregnancy rate	Yes, but in favor of day 2-3 transfers, compared to single blastocyst	1.58 (95% CI = 1.11-2.25)	Glujovsky et al., 2012 (Cochrane review)
Morpho-kinetics by time-lapse systems	Live birth	No	1.11 (95% CI = 0.45-2.73)	Armstrong et al., 2015 (Cochrane review)
	Clinical pregnancy	No	1.23 (95% CI = 0.96-1.59)	Armstrong et al., 2015 (Cochrane review)
Embryo metabolome in culture medium	Live birth	No	0.98 (95% CI = 0.74-1.29)	Vergouw et al., 2014 (Meta-analysis)

## References

[1] Ahlström A, Wikland M, Rogberg L (2011). Cross-validation and predictive value of near-infrared spectroscopy algorithms for day-5 blastocyst transfer.. Reprod Biomed Online.

[2] Armstrong S, Arroll N, Cree LM (2015). Time-lapse systems for embryo incubation and assessment in assisted reproduction.. Cochrane Database Syst Rev.

[3] Balaban B, Urman B, Isiklar A (2001). The effect of pronuclear morphology on embryo quality parameters and blastocyst transfer outcome.. Hum Reprod.

[4] Basile N, del Carmen NM, Bronet F (2014). Increasing the probability of selecting chromosomally normal embryos by time-lapse morphokinetics analysis.. Fertil Steril.

[5] Berger DS, Zapantis A, Merhi Z (2014). Embryo quality but not pronuclear score is associated with clinical pregnancy following IVF.. J Assist Reprod Genet.

[6] Brison DR, Houghton FD, Falconer D (2004). Identification of viable embryos in IVF by noninvasive measurement of amino acid turnover.. Hum Reprod.

[7] Butler SA, Luttoo J, Freire MO (2013). Human chorionic gonadotropin (hCG) in the secretome of cultured embryos: hyperglycosylated hCG and hCG-free beta subunit are potential markers for infertility management and treatment.. Reprod Sci.

[8] Campbell A, Fishel S, Bowman N (2013). Modelling a risk classification of aneuploidy in human embryos using non- invasive morphokinetics.. Reprod Biomed Online.

[9] Chavez SL, Loewke KE, Han J (2012). Dynamic blastomere behaviour reflects human embryo ploidy by the four- cell stage.. Nat Commun.

[10] Conaghan J, Cjhen AA, Willman SP (2013). Improving embryo selection using a computer-automated time-lapse imageanalysis test plus day 3 morphology: results from a prospective multicenter trial.. Fertil Steril.

[11] Cruz M, Garrido N, Herrero J (2012). Timing of cell division in human cleavage-stage embryos is linked withblastocyst formation and quality.. Reprod Biomed Online.

[12] Dahdouh EM, Balayla J, García-Velasco JA (2015). Impact of blastocyst biopsy and comprehensive chromosome screening technology on preimplantation genetic screening: a systematic review of randomized controlled trials.. Reprod Biomed Online.

[13] Edwards RG, Fishel SB, Cohen J (1984). Factors influencing the success of in vitro fertilization for alleviating human infertility.. J In Vitro Fert Embryo Transf.

[14] El-Toukhy T, Khalaf Y, Braude P (2006). IVF results: optimize not maximize.. Am J Obstet Gynecol.

[15] Fisch JD, Rodriguez H, Ross R (2001). The Graduated Embryo Score (GES) predicts blastocyst formation and pregnancy rate from cleavage-stage embryos.. Hum Reprod.

[16] Gardner DK, Leese HJ (1987). Assessment of embryo viability prior to transfer by the noninvasive measurement of glucose uptake.. J Exp Zool.

[17] Gardner DK, Leese HJ (1990). Concentrations of nutrients in mouse oviduct fluid and their effects on embryo development and metabolism in vitro.. J Reprod Fertil.

[18] Gardner DK, Lane M, Stevens J (2000). Blastocyst score affects implantation and pregnancy outcome: towards a single blastocyst transfer.. Fertil Steril.

[19] Gardner DK, Lane M, Stevens J (2001). Noninvasive assessment of human embryo nutrient consumption as a measure of developmental potential. Fertil Steril.

[20] Gardner DK, Wale PL, Collins R (2011). Glucose consumption of single post-compaction human embryos is predictive of embryo sex and live birth outcome.. Hum Reprod.

[21] Gardner DK, Wale PL (2013). Analysis of metabolism to select viable human embryos for transfer.. Fertil Steril.

[22] Giorgetti C, Terriou P, Auquier P (1995). Embryo score to predict implantation after in-vitro fertilization: based on 957 single embryo transfers.. Hum Reprod.

[23] Glujovsky D, Blake D, Farquhar C (2012). Cleavage stage versus blastocyst stage embryo transfer in assisted reproductive technology.. Cochrane Database Syst Rev.

[24] Goldman-Wohl DS, Ariel I, Greenfield C (2000). HLA-G expression in extravillous trophoblasts is an intrinsic property of cell differentiation: a lesson learned from ectopic pregnancies.. Mol Hum Reprod.

[25] González RR, Caballero-Campo P, Jasper M (2000). Leptin and leptin receptor are expressed in the human endometrium and endometrial leptin secretion is regulated by the human blastocyst. J Clin Endocrinol Metab.

[26] Goodman LR, Goldberg J, Falcone T (2016). Does the addition of time-lapse morphokinetics in the selection of embryos for transfer improve pregnancy rates? A randomized controlled trial.. Fertil Steril.

[27] Gott AL, Hardy K, Winston RM (1990). Noninvasive measurement of pyruvate and glucose uptake and lactate production by single human preimplantation embryos.. Hum Reprod.

[28] Guo XY, Jiang F, Cheng XJ (2013). Embryonic soluble human leukocyte antigen-G as a marker of embryo competency in assisted reproductive technology for Chinese women.. J Reprod Med.

[29] Houghton FD, Thompson JG, Kennedy CJ (1996). Oxygen consumption and energy metabolism of the early mouse embryo.. Mol Reprod Dev.

[30] Houghton FD, Hawkhead JA, Humpherson PG (2002). Noninvasive amino acid turnover predicts human embryo developmental capacity.. Hum Reprod.

[31] Hsu MI, Mayer J, Aronshon M (1999). Embryo implantation in in vitro fertilization and intracytoplasmic sperm injection: impact of cleavage status, morphology grade, and number of embryos transferred.. Fertil Steril.

[32] Jones GM, Trounson AO, Vella PJ (2001). Glucose metabolism of human morula and blastocyst-stage embryos and its relationship to viability after transfer.. Reprod Biomed Online.

[33] Juneau C, Franasiak J, Treff N (2016). Challenges facing contemporary preimplantation genetic screening. Curr Opin Obstet Gynecol.

[34] Hollywood K, Brison DR, Goodacre R (2006). Metabolomics: current technologies and future trends.. Proteomics.

[35] Kahraman S, Kumtepe Y, Sertyel S (2002). Pronuclear morphology scoring and chromosomal status of embryos in severe male infertility.. Hum Reprod.

[36] Katz-Jaffe MG, Schoolcraft WB, Gardner DK (2006). Analysis of protein expression (secretome) by human and mouse preimplantation embryos. Fertil Steril.

[37] Kirkegaard K, Svane AS, Nielsen JS (2014). Nuclear magnetic resonance metabolomic profiling of Day 3 and 5 embryo culture medium does not predict pregnancy outcome in good prognosis patients: a prospective cohort study on single transferred embryos.. Hum Reprod.

[38] Kotze D, Kruger TF, Lombard C (2013). The effect of the biochemical marker soluble human leukocyte antigen G on pregnancy outcome in assisted reproductive technology--a multicenter study.. Fertil Steril.

[39] Kovacs P (2014). Embryo selection: the role of time-lapse monitoring.. Reprod Biol Endocrinol.

[40] Leese HJ, Hooper MA, Edwards RG (1986). Uptake of pyruvate by early human embryos determined by a noninvasive technique.. Hum Reprod.

[41] Lipari CW, Garcia JE, Zhao Y (2009). Nitric oxide metabolite production in the human preimplantation embryo and successful blastocyst formation. Fertil Steril.

[42] Lopes AS, Greve T, Callesen H (2007). Quantification of embryo quality by respirometry.. Theriogenology.

[43] Ludwig M, Schöpper B, Al-Hasani S (2000). Clinical use of a pronuclear stage score following intracytoplasmic sperm injection: impact on pregnancy rates under the conditions of the German embryo protection law.. Hum Reprod.

[44] Manna C, Patrizi G, Rahman A (2004). Experimental results on the recognition of embryos in human assisted reproduction.. Reprod Biomed Online.

[45] Mastenbroek S, Twisk M, van der Veen F (2011). Preimplantation genetic screening: a systematic review and meta-analysis of RCTs.. Hum Reprod Update.

[46] Meseguer M, Herrero J, Tejera A (2011). The use of morphokinetics as a predictor of embryo implantation.. Hum Reprod.

[47] Mio Y, Maeda K (2008). Time-lapse cinematography of dynamic changes occurring during in vitro development of human embryos. Am J Obstet Gynecol.

[48] Montskó G, Zrínyi Z, Janáky T (2015). Noninvasive embryo viability assessment by quantitation of human haptoglobin alpha-1 fragment in the in vitrofertilization culture medium: an additional tool to increase success rate.. Fertil Steril.

[49] Nagy ZP, Sakkas D, Behr B (2008). Non-invasive assessment of embryo viability bymetabolomic profiling of culture media (’metabolomics’). Reprod Biomed Online.

[50] Nagy ZP, Jones-Colon S, Roos P (2009). Metabolomic assessment of oocyte viability.. Reprod Biomed Online.

[51] O’Donovan C, Twomey E, Alderman J (2006). Development of a respirometric biochip for embryo assessment.. Lab Chip.

[52] O’Leary T, Heindryckx B, Lierman S (2013). Derivation of human embryonic stem cells using a post-inner cell mass intermediate.. Nat Protoc.

[53] O’Neill C, Saunders M (1984). Assessment of embryo quality. Lancet.

[54] O’Neill C (2005). The role of paf in embryo physiology.. Hum Reprod Update.

[55] Ozawa M, Nagai T, Fahrudin M (2006). Addition of glutathione or thioredoxin to culture medium reduces intracellular redox status of porcine IVM IVF embryos, resulting in improved development to the blastocyst stage.. MolReprod Dev.

[56] Paszkowski T, Clarke RN (1996). Antioxidative capacity of preimplantation embryo culture medium declines following the incubation of poor quality embryos.. Hum Reprod.

[57] Payne D, Flaherty SP, Barry MF (1997). Preliminary observations on polar body extrusion and pronuclear formation in human oocytes using time-lapse video cinematography.. Hum Reprod.

[58] Picton HM, Elder K, Houghton FD (2010). Association between amino acid turnover and chromosome aneuploidy during human preimplantation embryo development in vitro.. Mol Hum Reprod.

[59] Ramu S, Acacio B, Adamowicz M (2011). Human chorionic gonadotropin from day 2 spent embryo culture media and its relationship to embryo development.. Fertil Steril.

[60] Renard JP, Philippon A, Menezo Y (1980). In-vitro uptake of glucose by bovine blastocysts.. J Reprod Fertil.

[61] Roudebush WE, Wininger JD, Jones AE (2002). Embryonic platelet-activating factor: an indicator of embryo viability.. Hum Reprod.

[62] Roussev RG, Coulam CB (2007). HLA-G and its role in implantation (review).. J Assist Reprod Genet.

[63] Rubio I, Galán A, Larreategui Z (2014). Clinical validation of embryo culture and selection by morphokinetic analysis: a randomized, controlled trial of the EmbryoScope.. Fertil Steril.

[64] Sallam HN, El Kafflsh DM, Ismail AA (2016). Embryo selection by measurement of soluble human leukocytic antigen-G levels in embryo culture medium in patients undergoing ICSI.. Fertil Steril.

[65] Salumets A, Hyden-Granskog C, Makinen S (2003). Early cleavage predicts the viability of human embryos in elective single embryo transfer procedures.. Hum Reprod.

[66] (2014). http://www.cdc.gov/art/ART2011.

[67] Seli E, Sakkas D, Scott R (2007). Noninvasive metabolomic profiling of embryo culture media using Raman and near- infrared spectroscopy correlates with reproductive potential of embryos in women undergoing in vitro fertilization.. Fert Steril.

[68] Seli E, Vergouw CG, Morita H (2010). Noninvasive metabolomic profiling as an adjunct to morphology for noninvasive embryo assessment in women undergoing single embryo transfer.. Fert Steril.

[69] Seli E, Bruce C, Botros LL (2011). Receiver operating characteristic (ROC) analysis of day 5 morphology grading and metabolomics Viability Score on predicting implantation outcome.. J Assist Reprod Genet.

[70] Scott RT, Seli E, Miller K (2008). Noninvasive metabolomic profiling of human embryo culture media using Raman spectroscopy predicts embryonic reproductive potential: a prospective blinded pilot study.. Fert Steril.

[71] Scott L, Alvero R, Leondires M (2000). The morphology of human pronuclear embryos is positively related to blastocyst development and implantation.. Hum Reprod.

[72] Shnizer S, Kagan T, Lanir A (2003). Modifications and oxidation of lipids and proteins in human serum detected by thermochemiluminescence.. Luminescence.

[73] Shoukir Y, Campana A, Farley T (1997). Early cleavage of in- vitro fertilized human embryos to the 2-cell stage: a novel indicator of embryo quality and viability.. Hum Reprod.

[74] Suzuki C, Yoshioka K, Sakatani M (2007). Glutamine and hypotaurine improves intracellular oxidative status and in vitro development of porcine preimplantation embryos.. Zygote.

[75] Tejera A, Herrero J, Viloria T (2012). Time-dependent O2 consumption patterns determined optimal time ranges for selecting viable human embryos.. Fertil Steril.

[76] Thompson JG, Partridge RJ, Houghton FD (1996). Oxygen uptake and carbohydrate metabolism by in vitro derived bovine embryos.. J Reprod Fertil.

[77] Trimarchi JR, Liu L, Porterfield M (2000). Oxidative phosphorylation-dependent and -independent oxygen consumption by individual preimplantation mouse embryos.. Biol Reprod.

[78] van Montfoort AP, Dumoulin JC, Kester AD (2004). Early cleavage is a valuable addition to existing embryo selection parameters: a study using single embryo transfers.. Hum Reprod.

[79] Van Royen E, Mangelschots K, De Neubourg D (1999). Characterization of a top quality embryo, a step towards single-embryo transfer.. Hum Reprod.

[80] Veeck LL (1991). Atlas of the human oocyte and early conceptus, Vol 2.

[81] Vercammen MJ, Verloes A, Van de Velde H (2008). Accuracy of soluble human leukocyte antigen-G for predicting pregnancy among women undergoing infertility treatment: meta- analysis.. Hum Reprod Update.

[82] Vergouw CG, Botros LL, Roos P (2008). Metabolomic profiling by near infrared spectroscopy as a tool to assess embryo viability: a novel, non-invasive method for embryo selection.. Hum Reprod.

[83] Vergouw CG, Botros LL, Judge K (2011). Non-invasive viability assessment of day-4 frozen-thawed human embryos using near infrared spectroscopy.. Reprod Biomed Online.

[84] Vergouw CG, Heymans MW, Hardarson T (2014). No evidence that embryo selection by near-infrared spectroscopy in addition to morphology is able to improve live birth rates: results from an individual patient data meta-analysis.. Hum Reprod.

[85] Wiener-Megnazi Z, Shiloh H, Avraham L (2011). Oxidative parameters of embryo culture media may predict treatment outcome in in vitro fertilization: a novel applicable tool for improving embryo selection.. Fertil Steril.

[86] Wong CC, Loewke KE, Bossert NI (2010). Non-invasive imaging of human embryos before embryonic genomeactivation predicts development to the blastocyst stage.. Nat Biotechnol.

[87] Xiao-Yan C, Jie L, Dang J (2013). A highly sensitive electro- chemiluminescence immunoassay for detecting human embryonic human chorionic gonadotropin in spent embryo culture media during IVF-ET cycle.. J Assist Reprod Genet.

[88] Zollner U, Zollner KP, Hartl G (2002). The use of a detailed zygote score after IVF ICSI to obtain good quality blastocysts: the German experience.. Hum Reprod.

